# Identification of sheep lncRNAs related to the immune response to vaccines and aluminium adjuvants

**DOI:** 10.1186/s12864-021-08086-z

**Published:** 2021-10-28

**Authors:** Martin Bilbao-Arribas, Endika Varela-Martínez, Naiara Abendaño, Damián de Andrés, Lluís Luján, Begoña M. Jugo

**Affiliations:** 1 Department of Genetics, Physical Anthropology and Animal Physiology, Faculty of Science and Technology, University of the Basque Country (UPV/EHU), Leioa, Spain; 2 Animal Health Department, Institute of Agrobiotechnology (IdAB, CSIC-Government of Navarra), Navarra, Spain; 3 Department of Animal Pathology, Instituto Universitario de Investigación Mixto Agroalimentario de Aragón (IA2), University of Zaragoza, Zaragoza, Spain

**Keywords:** LncRNAs, *Ovis aries*, Vaccine, Adjuvants, Aluminium, Systems biology, Immune response, Co-expression, RNA-seq

## Abstract

**Background:**

Long non-coding RNAs (lncRNAs) are involved in several immune processes, including the immune response to vaccination, but most of them remain uncharacterised in livestock species. The mechanism of action of aluminium adjuvants as vaccine components is neither not fully understood.

**Results:**

We built a transcriptome from sheep PBMCs RNA-seq data in order to identify unannotated lncRNAs and analysed their expression patterns along protein coding genes. We found 2284 novel lncRNAs and assessed their conservation in terms of sequence and synteny. Differential expression analysis performed between animals inoculated with commercial vaccines or aluminium adjuvant alone and the co-expression analysis revealed lncRNAs related to the immune response to vaccines and adjuvants. A group of co-expressed genes enriched in cytokine signalling and production highlighted the differences between different treatments. A number of differentially expressed lncRNAs were correlated with a divergently located protein-coding gene, such as the OSM cytokine. Other lncRNAs were predicted to act as sponges of miRNAs involved in immune response regulation.

**Conclusions:**

This work enlarges the lncRNA catalogue in sheep and puts an accent on their involvement in the immune response to repetitive vaccination, providing a basis for further characterisation of the non-coding sheep transcriptome within different immune cells.

**Supplementary Information:**

The online version contains supplementary material available at 10.1186/s12864-021-08086-z.

## Background

Aluminium-containing adjuvants have been used for nearly a century now both in livestock and in humans since their discovery in the early 20th century [1]. Aluminium salts such as aluminium hydroxide or aluminium phosphate are the most common compounds used as adjuvants to increase the immunogenicity of vaccines. Despite their good safety record, the mechanism of action of these adjuvants has not been fully characterised [2]. Current hypotheses include the activation of the NLRP3 inflammasome, release of DNA and uric acid danger signals, activation of the Syk-PI3K pathway and others [3], but aluminium adjuvants will most likely exert their function by multiple of these and more factors. An analysis of gene expression and proteome of Al(OH)3 treated monocytes revealed two new pathways activated by the adjuvant – IFNβ signalling and HLA class I antigen processing and presentation – and signatures of both Th1 and Th2 immune response [4].

Systems vaccinology approaches, thus application of systems biology during the development of vaccines, can be used to study the mechanism of action of adjuvants, the immune responses induced by them or, more practically, to improve the quality of vaccines [5]. Transcriptional profiles of tissues in vivo provide valuable information on the behaviour of genes after exposure to vaccines or adjuvants, including the study of non-coding transcripts, which are becoming more relevant in immunology. Recent studies have shown that lncRNAs in blood cells participate in the immune response to vaccines since the expression of several long non coding RNAs (lncRNAs) change after vaccination and correlate to antibody production [6]. In the context of sheep research, studies profiling the transcriptomic response to vaccines are scarce [7, 8], with almost none of them focusing on lncRNAs or vaccine adjuvants [9]. In human, transcriptomic studies have been used for the dissection of adjuvant mechanism of action [10, 11], and only one murine study analysed the lncRNAs induced by aluminium salts [12].

Long non-coding RNAs, defined as transcripts longer than 200 nucleotides that lack protein-coding capability and are consistently transcribed, show spatiotemporal-specific expression patterns that highlight the diverse processes in which they are involved [13]. In immune cells lncRNAs are expressed in a very cell-specific and dynamic way, even within lineages of the same cell types [14–16] and this cell-type specificity seems to be conserved among species [17]. Because of this, it is becoming apparent that lncRNAs are involved in immune system cell gene expression regulation, which should be finely regulated for the generation of a correct immunity and to avoid autoimmune responses.

Thousands of lncRNAs that may have important roles in immune processes are being described every year, but most of them remain functionally uncharacterised, especially in particular in non-human species. Many of them might simply be transcriptional noise, but several other seem to be functional [18]. In a recent collaborative project, more than the 25 % of studied lncRNAs were found to affect the molecular phenotype of human fibroblasts [19]. LncRNAs do not have a single molecular mechanism. Many of the described lncRNAs function by acting as scaffolds via interactions with DNA, RNA and proteins [20]. Sometimes the act of transcription itself has a local functional output [21], which could explain the low sequence conservation of some lncRNAs. The functions of lncRNAs are generally classified as cis or trans, depending if the effect happens in a local or distant genomic region [22].

In this work, we analysed RNA sequencing data from a previous study carried out in our lab, in which it was characterised the effect of Al hydroxide adjuvant on the immune response to vaccination was characterised in a long-term experiment using sheep as a model [23] for the profiling of novel lncRNAs. We identified novel lncRNAs in sheep peripheral blood mononuclear cells (PBMCs), a subset of blood cells consisting of multiple immune cells including lymphocytes, monocytes and dendritic cells that is broadly used in infectious disease and vaccine research to get a global view of molecular and cellular events during the development of an immune response [24]. We assessed their expression kinetics along with protein coding genes (PCGs) and miRNAs by differential expression analysis and detection of co-expressed gene modules.

## Results

### Identification and classification of lncRNAs

**Fig. 1:**
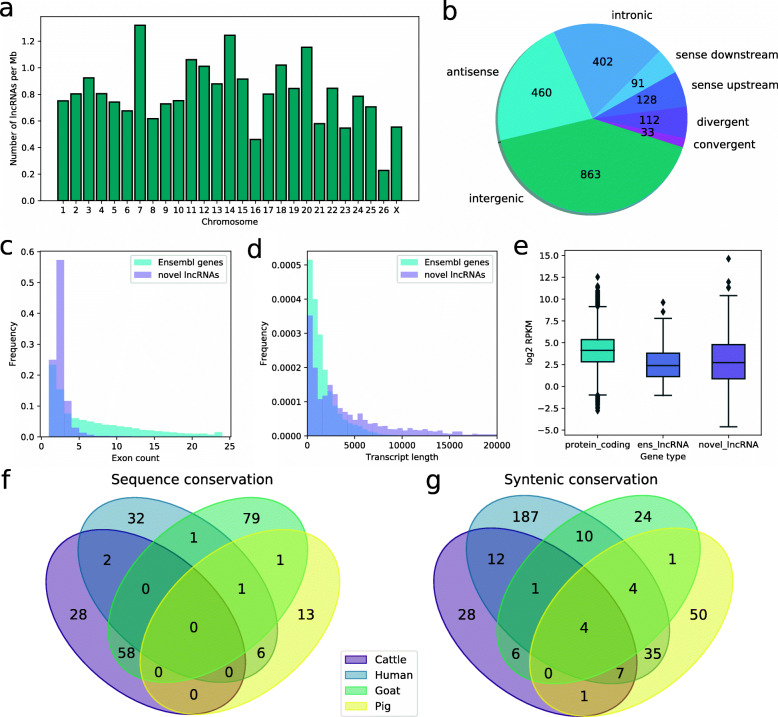
General characteristics of the novel lncRNAs. **a** LncRNA density per chromosome. **b** Classification of detected candidate lncRNAs by relative location to the closest annotated gene. **c** Exon number distribution in novel lncRNAs and annotated genes. **d** Transcript length distribution in novel and annotated genes. **e** Mean expression of protein coding genes, annotated lncRNAs and novel lncRNAs. **f** Novel lncRNAs conserved at sequence level comparing with selected Ensembl annotations. **g** Novel lncRNAs with conserved synteny in selected Ensembl annotations

Unknown intergenic, intronic and antisense transcripts were filtered by length and exon count, reducing the list of potential lncRNAs from 10,340 to 4899. Transcripts were further assessed for protein coding potential, reducing the list to 2284 transcripts. These 2284 lncRNA transcripts were defined as the novel set of lncRNAs (Additional file 1). Despite their different approaches, CPAT, CPC2 and HMMER filtered the transcripts with high overlap, with 72 %, 56 % and 68 % of the predictions, respectively, included in the final set. Candidate lncRNAs were evenly distributed across chromosomes, with larger ones containing more transcripts (Fig. 1a). Due to the 2000 nucleotide length threshold for monoexonic transcripts, 2-exon transcripts were the most numerous (Fig. 1c) and showed a wider range of lengths than annotated genes (Fig. 1d). Single-exon transcripts were mostly shorter than 5000 nucleotides while transcripts with more than 2 exons had diverse lengths. As for the classification of lncRNAs based on their relative location to their closest genes, the intergenic class was the most numerous (38 %), followed by antisense (20 %) and intronic (18 %) transcripts (Fig. 1b). Among those intergenic transcripts very close to an annotated gene (distance < 5 kb), we found 112 (5 %) divergent lncRNAs, which are interesting because they could share the promoter with its flanking gene. PCGs were more highly expressed than lncRNAs, and mean expression levels of novel lncRNAs and annotated lncRNAs were similar (Fig. 1e). These results are in concordance with some previous studies, even if due to a lack of a standardised workflow different results are obtained depending on the analyses done and applied thresholds.

We compared our shortlisted lncRNAs in PBMCs with other works in sheep that also identify novel lncRNAs by searching for transcripts that share a TSS, defined as the first transcribed nucleotide, and that are transcribed in the same direction. In brain tissue of animals from the same experiment [9] 315 transcripts (14 %) shared a TSS. However, examining other works with available annotation of new lncRNA, small numbers of transcripts present in other tissues were found. Just 33 transcripts (1.44 %) shared a TSS with a lncRNA from a multi-tissue catalogue [25] and 56 (2.45 %) with lncRNAs from pituitary gland [26].

### Conservation in terms of sequence and synteny

Evolutionary conservation of lncRNAs can be an indicator of function. In this way, having orthologues strengthens the evidence on sequenced transcripts, even more if the lncRNA has already been characterised in other species. As expected because of the nature of lncRNAs, few sequences had matches with other species (Fig. 1f, Additional file 2). The highest number of conserved sequences were in goat (6.67 %), then cattle (4.28 %), human (2.09 %) and pig (1.07 %). The human conserved lncRNAs included several functionally characterised lncRNAs such as CHASERR, CYTOR, CCDC26 or FTX. Just eight transcripts (0.35 %) had confident matches with cattle NONCODE sequences. Note that 185 annotated sheep lncRNAs (9.96 % of all annotated lncRNAs) were also detected above the minimum expression threshold in PBMCs.

In terms of gene order, more transcripts appeared to be located in conserved regions (Fig. 1g, Additional file 2), some even showing short alignments with annotated lncRNAs in the same region. We could perform the synteny analysis with roughly half of the novel lncRNAs, those surrounded with PCGs no more than 500 kb away. The 2.55 % of novel sheep lncRNAs shared the same syntenic location with an annotated cattle lncRNA, and 2.19 % with goat lncRNAs, a number that was higher in human (11.36 %). Both sequence and conservation analyses are biased due to the vast quantity of lncRNAs annotated in the human genome (17,959) comparing with other livestock species, whose lncRNA repertoire is not fully annotated and also diverge in the quantity of lncRNA genes (1858 in sheep, 2705 in goat, 1480 in cattle and 6790 in pig). Because of this, when performing the same analysis with the 22,227 cattle NONCODE lncRNAs 9.93 % of novel lncRNAs show syntenic conservation. Few of these lncRNAs with shared syntenic location showed short highly conserved alignments.

### Expression analysis

**Fig. 2:**
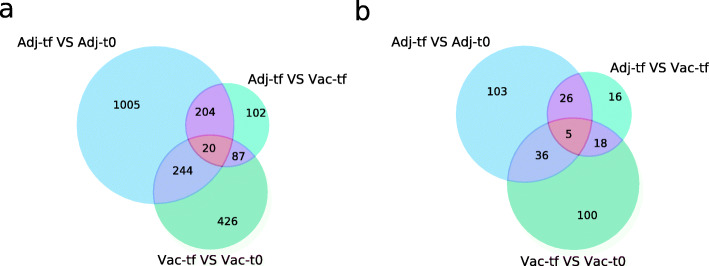
Venn diagrams of differential expression of coding and lncRNA genes. **a** Total differentially expressed genes. **b** Differentially expressed novel lncRNA genes. Comparisons were made between time points in vaccinated animals (Vac-tf vs. Vac-t0), between time points in adjuvant-only animals (Adj-tf vs. Adj-t0) and between the treatments at the end of the experiment (Adj-tf vs. Vac-tf)

In order to profile the expression of lncRNAs in the presence of aluminium adjuvants, differential expression was tested between treatment groups. The analysis was made with all annotated genes plus the newly identified candidate lncRNAs. In the same fashion as annotated genes [23], there were less DE lncRNAs in the comparison between both treatments at the end of the experiment than between each treatment at the start and end of the experiment (Fig. 2, Additional file 3). 170 lncRNAs were differentially expressed in the Adj-t0 vs. Adj-tf comparison (19 annotated and 151 candidate lncRNAs). 159 lncRNAs were differentially expressed in the Vac-t0 vs. Vac-tf comparison (11 annotated and 148 candidate lncRNAs). 65 lncRNAs were differentially expressed in the Adj-tf vs. Vac-tf comparison (4 annotated and 61 candidate lncRNAs). The expression divergence is clear when comparing time-points, while treatment-wise changes are more subtle. We found that five of the DE novel lncRNAs are conserved between sheep and human. The divergent MSTRG.24,028 lncRNA is downregulated in the Adj-t0 vs. Adj-tf comparison and is homologous to the human OTUD6B-AS1 lncRNA, which has been recently linked to regulation of apoptosis [27].

**Fig. 3:**
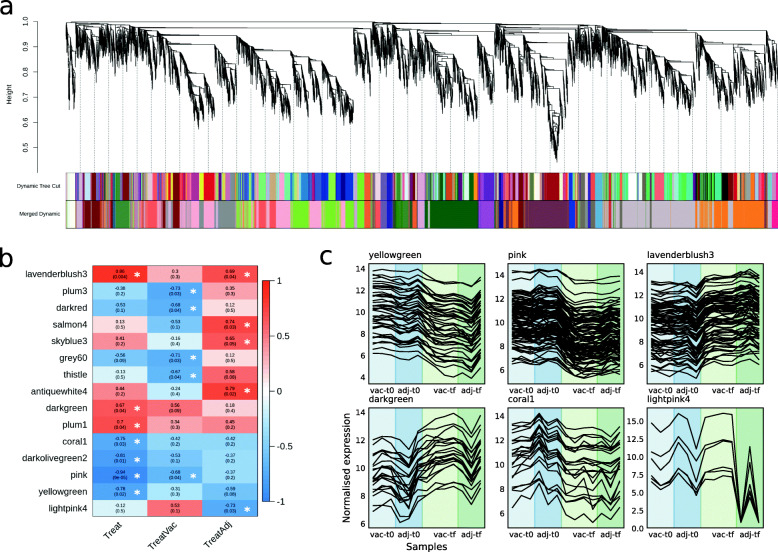
WGCNA co-expression analysis results. **a** Gene dendrogram obtained by average linkage hierarchical clustering. The colour bars show the module assignment before and after modules with similar expression profiles were merged. **b** Module-trait associations. Each row corresponds to a module eigengene, while columns correspond to a trait (both treatments together, vaccine and adjuvant-only). Only modules associated with at least one trait are shown. **c** Expression profiles of hub genes of modules correlated with at least one trait and that are enriched in some GO terms

A gene co-expression network was constructed with the same genes used for differential expression. This analysis provides valuable information about along which genes are the candidate lncRNAs expressed, and in this way, predicting their putative functions by guilt-by-association. Genes with similar expression patterns were clustered in 32 modules ranging from 39 to 1956 genes (Fig. 3a, Additional file 4). We searched for significant correlations among module eigengenes, the principal component of the genes in the module that depicts its dominant trend, and treatment parameters. 15 modules were correlated with at least one treatment: 5 modules with the adjuvant treatment, 5 modules with the vaccine treatment and 7 modules with both treatments taken together as a single group (Fig. 3b).

As for the module membership of candidate lncRNAs, most modules were made of both PCGs and lncRNAs, although in differing proportions. The five modules with more than 1000 genes had many co-expressed lncRNAs, while some small modules were only composed of PCGs. Integrating DE and co-expression analysis, 17 modules had DE genes within them, most of them belonging to the comparisons between time points.

Modules were characterized by gene enrichment analysis and showed involvement in distinct biological processes (Additional file 4). Some modules were not enriched in any term, mainly the smaller ones, and others were enriched in cell cycle functions or general metabolic functions. Two modules (coral1 and lightpink4) were clearly linked to the immune response with functions related to cytokines, immune cell differentiation and response to stress and external stimuli.

### Treatment-correlated co-expression modules

Modules with significant correlations with a treatment variable were selected for further analysis, since lncRNAs in those modules are probably responding to the vaccine or adjuvants and many of them are differentially expressed. Modules whose eigengene is correlated with the treatment variable should reveal information about the general effect of aluminium on the immune response and modules whose eigengene is correlated with one of the treatments should highlight the differences between them. The expression profiles of the hub genes within each significantly correlated module show the trend of those modules across treatment groups (Fig. 3c).

**Fig. 4:**
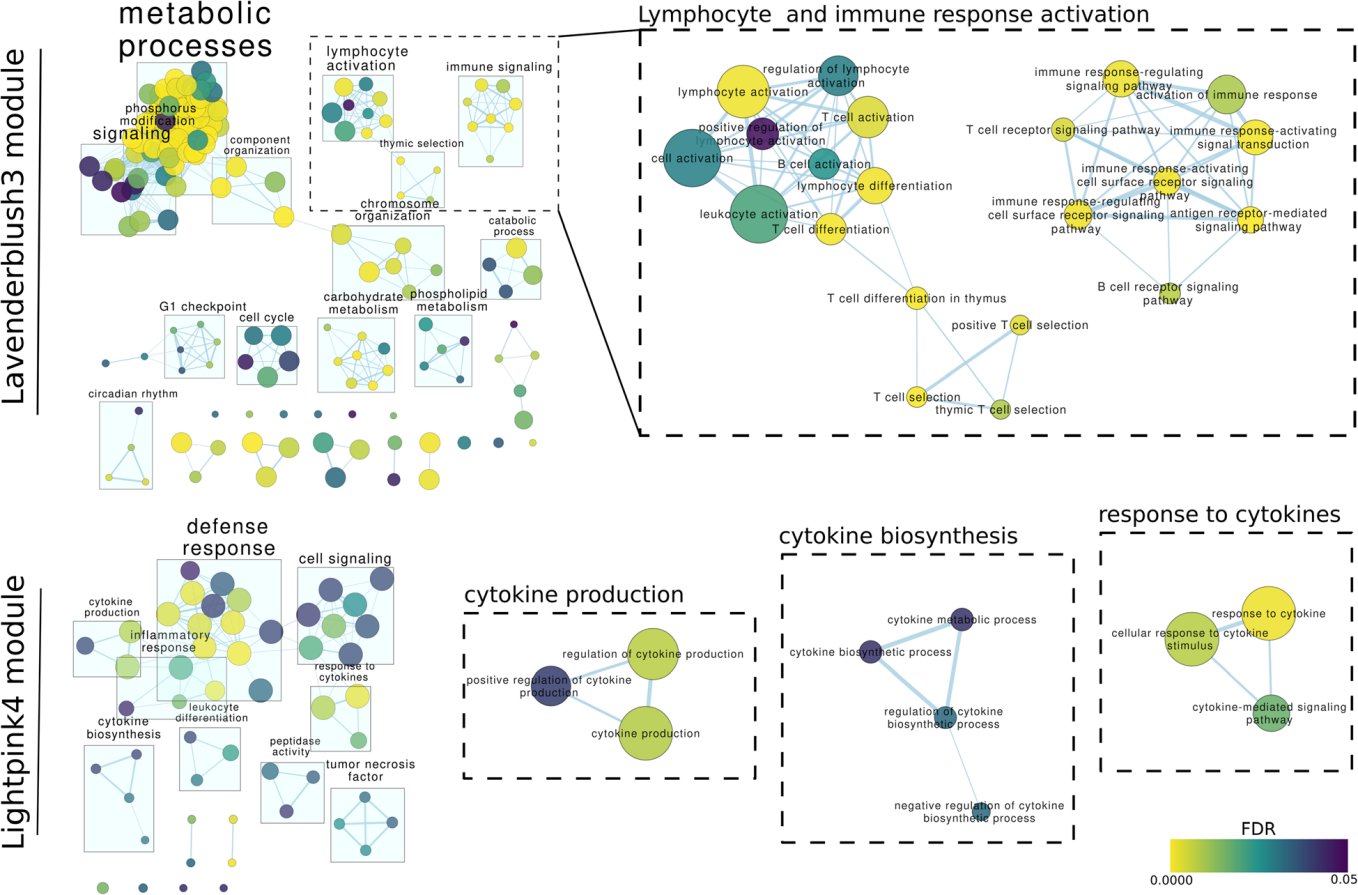
Networks of enriched GO biological process functions in two trait-correlated modules: Lavenderblush3 and Lightpink4. Nodes represent GO biological process terms. Nodes are coloured by false discovery rate (FDR) and their size represents the number of genes in the module belonging to the term. Edge width represents the number of shared genes between two terms

Among the modules correlated with both treatments at the same time, the pink module had the strongest correlation (9e-0.5 p value) and was enriched in DNA repair, methylation and general metabolic processes. Coral1 module was enriched in diverse processes such as immune response, T-helper cell functions (Th17 specifically), inflammation, cell motility or proliferation; all of these in concordance with a general response of the immune system. The yellowgreen module included genes related to the respiratory chain and cell cycle. Lavenderblush3 is highly correlated with the treatment variable, independent of its composition, and it is enriched in immune response activation, lymphocyte activation, cell cycle and metabolic processes (Fig. 4).

The most prominent module correlated with a specific treatment variable was lightpink4, negatively correlated with the adjuvant treatment, suggesting a tendency for lower expression in the adjuvant group (Fig. 3c). It is enriched in responses to external stimuli, cytokines and differentiation of various immune cells (Fig. 4); and its expression seems to be driven by many DE genes in the Adjuvant tf vs. Vaccine tf comparison. Besides, this module includes marker genes of classical monocytes (CD14, S100A12, S100A8) and non-classical monocytes (FCGR3A) [28], possibly indicating a reduction in the monocyte lineage fraction of PBMCs in the Adjuvant tf group. S100A12 and S100A8 are known to be highly expressed in bone marrow-derived macrophages of sheep and other mammals [29]. Other abundant genes in this module are those involved in cytokine production and reception (e.g. IL6R, IL1R1, IL1R2, IRF1, PTGER4, MYD88, IL17RC, OSM, IL15RA, IL4R, CXCR1, CSF2RB, CSF3R). The genes CXCR1, CSF2RB and CSF3R are hub genes of this module.

### Expression of nearby PCGs and lncRNAs

Correlated lncRNA-PCG pairs were identified as a way of inferring potential cis regulation. In the RNA-seq dataset, 348 lncRNAs-PCG pairs showed correlations above the applied threshold. Most of the involved lncRNAs were sense intronic, sense upstream or sense downstream of their correlated gene, but there were 24 antisense lncRNAs, 9 divergent lncRNAs and 34 intergenic lncRNAs.

**Fig. 5:**
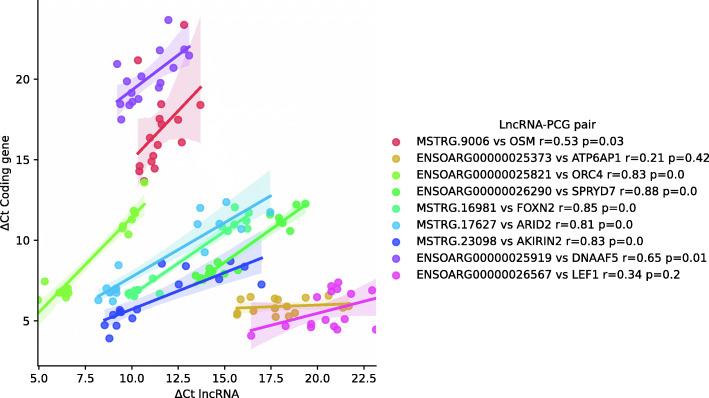
Expression correlations between selected lncRNA and protein coding gene (PCG) pairs assessed by RT-qPCR. Gene expression correlations were performed with efficiency corrected ΔCt values and Spearman’s rank correlation

Relative expression levels of 10 pairs of correlated lncRNAs and PCGs were measured by RT-qPCR in order to validate their coordinated expression. Six differentially expressed lncRNAs and 4 non-differentially expressed lncRNAs were selected. Half of the selected lncRNAs were classified as divergent (MSTRG.9006, ENSOARG00000025373, MSTRG.17,627, MSTRG.23,098, ENSOARG00000025919), and there were two sense (ENSOARG00000026290, MSTRG.16,981), two intergenic (ENSOARG00000025821, ENASOARG00000026567) and one antisense (ENSOARG00000026120) lncRNAs. All except for the antisense one were amplified, including those that are unannotated in Ensembl and are predicted in this study. 7 out of 9 amplified lncRNAs (78 %) showed significant correlations with their corresponding PCG (Fig. 5).

Among the studied pairs, some are interesting due to their relationship with the immune system: The gene ENSOARG00000006353, an orthologue of human and murine OSM gene, encodes for a cytokine secreted by monocytes/macrophages and T-lymphocytes, and is involved in haematopoiesis and inflammation [30]. It is divergently located to the novel monoexonic MSTRG.9006 lncRNA and both of them are differentially expressed in the vaccinated group. Another immune related gene, the transcription factor FOXN2, is correlated with the lncRNA MSTRG.16,981 located sense upstream of it and is differentially expressed in the adjuvant group. Besides, three novel lncRNAs, which were not differentially expressed in the RNA-seq dataset, showed robust correlations with coding genes ARID2, AKIRIN2 and DNAAF5 in a divergent position.

### Novel lncRNAs as miRNA sponges

**Fig. 6:**
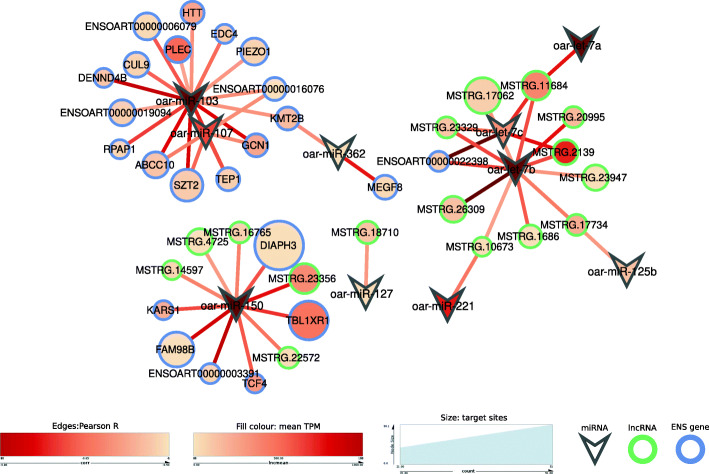
Network of miRNA sponge candidates. Significant negative Pearson correlations between miRNAs and target genes are depicted as edges. Size of target genes reflects the amount of target sites for a miRNA. Inner colours represent TPM expression and edge colours Pearson correlation strength (r)

Some lncRNAs could be acting as miRNA sponges due to their high quantity of predicted miRNA binding sites. One hundred lncRNAs, 2 annotated lncRNAs and 69 PCGs had more than 20 predicted target sites for at least one expressed miRNA. 22 miRNAs were involved in those interactions. Assuming that miRNAs downregulate the expression of their targets, we calculated the expression correlations between them. 16 novel lncRNAs and 26 PCGs showed significant negative correlations with a miRNA (Fig. 6). The miRNAs that target most lncRNAs are oar-let-7b and oar-miR-150. The highly expressed let-7b was upregulated in the Adj-t0 vs. Adj-tf comparison [23]. The other miRNA, oar-miR-150, was also one of the most expressed in the miRNA dataset of the same experiment [23].

## Discussion

Mining lncRNAs from RNA-seq data allows the detection of large amounts of transcripts that could be classified as candidate lncRNAs. Although there was an overlap between *a priori* transcriptionally different tissues such as brain [9] and PBMCs of the same experimental animals, the identified lncRNAs were mostly tissue-specific, as few of them were present in other studies in sheep The newly identified lncRNAs shared similar features with those previously found in other mammal studies: lower expression than PCGs, fewer exons, limited sequence conservation and a majority of intergenic transcripts. For instance, using a multi-tissue expression dataset, 12,296 and 2657 lncRNAs with intergenic location mainly were identified in sheep and goat [25]. In a developmental tissue dataset from seven species, mostly species-specific lncRNAs were found [31]. Other sheep works analysed lncRNAs within a specific functional RNA-seq dataset and identify lncRNAs with similar characteristics [26, 32–34].

Apart from a set of highly conserved and functionally characterised lncRNAs [35], lncRNAs show low sequence conservation. Hence, some may be functionless, function by the act of transcription itself [21, 36, 37], like the bidirectionally transcribed class of eRNAs [38], or have short functional elements that escape common conservation analyses. Some of the highly conserved lncRNAs identified in this work have been experimentally tested in humans. For instance, Chaserr (LINC01578), that negatively regulates its adjacent gene CHD2, to tune its expression [39], and lnc-sox5, that promotes the expression of IDO1, which modulates T-cell behaviour [40].

A large fraction of annotated lncRNAs are divergent lncRNAs, originated upstream of an specific gene and regulated by a bidirectional promoterso they often show expression correlations with their adjacent gene, which can imply a regulatory relationship [41, 42]. Based on this statement, the function of unknown lncRNAs may be inferred from their relationship with adjacent genes [43]. We found 112 lncRNAs which could be classified as divergent. in the RNA-seq dataset. Five divergent lncRNA-PCG pairs with significant correlations were tested also by RT-qPCR. Among those pairs, the gene coding for the OSM cytokine was correlated with a 3 kb long monoexonic lncRNA not annotated in sheep. Both genes were upregulated in the vaccinated group of animals. Although pending of functional studies, this could be an example of a bidirectional promoter, known to be stronger than regular promoters [44], that increases transcription of a PCG.

To predict functions of lncRNAs, prioritise candidates and discern their transcriptional regulatory programmes a coexpression analysis network was performed, assuming that lncRNAs related to known genes are involved in the same processes or pathways. Thus, we hypothesise that differentially expressed lncRNAs co-expressed with known immune genes are more likely to be involved in immune response functions,. The gene set enrichments of co-expression modules responding to both treatments pointed to aluminium-induced inflammation, while the modules responding only to vaccines or aluminium adjuvants alone highlighted the effect of adding antigens to the adjuvant preparation, as illustrated by an immune gene-rich module with several genes involved in cytokine production and reception, and monocyte markers. This module included many novel lncRNAs, including the one divergently located to the OSM cytokine gene.

Lastly, the data sets were analyzed to investigate the interaction between two regulatory elements, lncRNAs and miRNAs. The miRNAs that target most lncRNAs were oar-let-7b and oar-miR-150. The highly expressed let-7b, being a regulator of innate immune response genes and inflammation activation [45, 46] was upregulated in the adjuvant inoculated animals [23].The second miRNA, oar-miR-150, was also one of the most expressed in the dataset [23]. It is thought to be important in the adaptive immune response due to its high expression in lymphocytes and its upregulation after vaccination [47, 48]. Thus, these lncRNAs could act as sponges by sequestrating miRNAs involved both in the innate and adaptive immune responses.

Future work should focus on annotating non-coding genes in specific immune cell types combining with functional experiments.

## Conclusions

The lncRNA transcriptome of sheep PBMCs after multiple vaccination or adjuvant-only inoculations was analysed. More than 2000 novel lncRNAs were found, a small proportion of them being conserved across close species. Some of those lncRNAs could be involved in the immune response to vaccination and could regulate nearby immune genes although experimental work should be performed to confirm their potential regulatory functions. Moreover, both treatments induced lncRNA-containing co-expression modules, highlighting their immune response signature. At last, some lncRNAs seem to act as sponges for 2 miRNAs involved in innate and adaptive immune responses. In this case, advances in systems vaccinology can shed light on the mechanism of action of aluminium salt adjuvants, and help to understand the overall immune response to vaccines.

## Methods

### Experiment design and sequencing data

 Raw data from a previous RNA-seq experiment performed by our group was analysed [23] for the detection of novel lncRNAs. All the animals used in this study were neutered male lambs of the same age without any vaccination before the experiment. The information regarding experimental design was included in [23]. In short, 14 Rasa Aragonesa lambs were divided in two treatment groups, one receiving commercial vaccines (Vac group) and the other only Alhydrogel aluminium hydroxide (Adj group), and were kept under controlled conditions for 475 days. During that time animals followed an inoculation schedule with commercial vaccines or Alhydrogel® only.

RNA was extracted from peripheral blood mononuclear cells (PBMCs) of three animals of each group at the beginning (t0) and at the end (tf) of the treatment. Ribosomal RNA-depleted total RNA was sequenced in a HiSeq2000 platform with a mean sequencing depth of 70 million and 2 × 75 nucleotide paired-end reads at CNAG (Centro Nacional de Análisis Genómico, Barcelona, Spain).

### Alignment, mapping and transcriptome assembly

Quality filtering, alignment and count estimates of annotated genes was made as previously [23] and using the same parameters. In short, adaptor sequence removal and quality filtering was performed with Trimmomatic v0.36 [49], reads were mapped to the sheep genome assembly Oar_v3.1 with STAR v2.5.2b [50] and quantification of the reference transcriptome was performed with featureCounts v1.5.0-p1 [43]. For the detection of non-annotated transcripts, like most lncRNAs, it is necessary to reconstruct the transcriptome. StringTie [51] assembler was run on each sample with the reference annotation from Ensembl 95 (Oar_v3.1) and, in order to obtain a non-redundant set of transcripts, the –merge option was applied afterwards. Then, StringTie was once again applied on each sample, but with the new GTF transcript file obtained in the previous step in order to estimate transcript abundances.

### Identification of candidate lncRNAs

GffCompare [52] software was used to classify all transcripts based on their location relative to the reference annotation. Potential lncRNAs were selected among those transcripts classified as unknown intergenic (u), fully contained within a reference intron (i) and in the opposite strand of a reference gene (x), since there is not enough evidence for other overlapping transcripts, which could arise due to errors or background noise. Potential lncRNAs were filtered by length and coding potential. First, multiexonic transcripts of less than 200 nucleotides and single-exon transcripts of less than 2000 nucleotides were filtered out. Secondly, three approaches were followed to assess the capability of the transcripts to code for proteins: Coding Potential Calculator 2 (CPC2) is a machine learning based program with a species-neutral model able to classify coding and non-coding sequences [53]. Coding-Potential Assessment Tool (CPAT) is another machine learning based program that we trained and selected the classification threshold following authors’ instructions using available bovine coding and non-coding sequences [54]. HMMER 3.1b2 [55] was used to detect Pfam protein domains in our potential lncRNAs, which were translated into the three possible frames. Transcripts classified as non-coding by CPC2 and CPAT and without protein domains detected by HMMER in any frame were selected as lncRNAs.

Each of the novel lncRNAs was classified based on its position relative to its closest gene. For parsing and classification we used custom Python scripts, including the BEDTools python implementation to get the closest genes (https://github.com/daler/pybedtools). Transcription start sites (TSSs) were defined as the start or stop nucleotides, depending on strandness. Seven categories or classes were defined: (1) antisense, for those transcripts overlapping a gene in the opposite strand; (2) intronic, for transcripts fully contained within an intron; (3) intergenic, for lncRNAs at least 5 kb away from any known gene; (4) divergent, with TSSs within 5 kb and in the opposite strand; (5) convergent, with transcription stops within 5 kb and in the opposite strand; (6) sense upstream, located less than 5 kb upstream of a gene and in the same strand; and (7) sense downstream, located less than 5 kb downstream of a gene and in the same strand.

### Sequence and synteny conservation

In order to find sequence level conservation of candidate lncRNAs, standalone Blast searches against the lncRNAs annotated in Ensembl Release 101 of four species: goat, cattle, pig and human. We libraries with lncRNA cDNA sequences for each species. We also downloaded cattle transcript sequences from NONCODE. Accounting for the low sequence conservation expected in lncRNAs, the threshold for identity was set to 50, the minimum length of the query sequence to half of the target’s length, E-value of 1 × 10 − 3 and query coverage of 50 %.

Synteny conservation, that is, the preservation of co-localisation of genes between different species, has been proposed as a way to deal with the low sequence conservation in lncRNAs. We downloaded from Ensembl BioMart (release 101) a custom dataset of all sheep (Oar v3.1) PCGs and their Ensembl-defined orthologues for goat (ARS1), cattle (ARS-UCD1.2), pig (Sscrofa11.1) and human (GRCh38). LncRNA annotations and cDNA sequences were also downloaded from Ensembl. Then, using a custom python script, we got the two upstream and downstream flanking orthologues for each lncRNA in the three species, which had to be located no more than 500 kb apart from it. Each sheep lncRNA was compared with all other lncRNAs. The minimum number of shared orthologues was set to two, these being the first flanking genes, and each pair of lncRNAs was scored as in the Ensembl Gene Order Conservation score. If the lncRNA was conserved in terms of synteny, an alignment was done between the novel sheep lncRNA transcript and the longest transcript of the other species’ gene with the Needleman-Wunsch global pairwise alignment from EMBOSS and the longest stretch of consecutive identical nucleotides in the alignment was calculated. It is thought that even if complete sequence conservation is not the most common in lncRNAs, small functional sequences could be conserved. The analysis was also performed with the set of cattle lncRNAs in NONCODE.

### Differential expression

The gene level expression matrix was built by keeping only the raw counts of novel lncRNAs obtained from StringTie and the count estimates of annotated genes. Before differential expression, SVA package [v3.26.0] [56] was applied to account for a known batch effect observed in the PCA analysis. After normalisation and removing of lowly expressed genes, three packages were used for differential expression: DESeq2 [57], limma [58] and edgeR [59]. Testing design included treatment, time, animal and SVA covariates, and differences were tested for the interaction of time and treatment. Thus, comparisons were made between the time points in both treatments (Vac tf vs. Vac t0 and Adj Tf vs. Adj t0) and between the treatments at the end of the experiment (Adj Tf vs. Vac Tf). The differentially expressed genes (DEGs) were selected from the intersection of the three tools of those genes with an adjusted p-value (using the Benjamini-Hochberg method) of < 0.05 and a log2 fold change (log2FC) value of > 1.

### Gene co-expression analysis

A weighted gene co-expression network analysis was performed using the WGCNA [v1.63] R package [60]. The similarity matrix was constructed from normalised expression data using the biweight midcorrelation, a correlation more robust against outliers. Next, the adjacency matrix was defined by raising the similarity matrix to a power β = 18, the minimum value required to get a scale-free topology network in our data. Modules, clusters of interconnected genes, were defined by performing a hierarchical clustering on the topological overlap measure. The minimum module size was set to 30 and modules with similar expression profiles were merged.

Once modules were defined, we looked for correlations with the treatment groups by dichotomising the groups in different combinations: samples at the beginning against samples at the end of the experiment (Treat variable), vaccine samples at the end against all other samples (TreatVac) and adjuvant samples at the end against all other samples (TreatAdj). For that purpose, Pearson correlations were generated for all pairwise comparisons of the module eigengene expression values and the treatment parameter. The eigengene is used to summarise each module with its first principal component. p-values were corrected by FDR (q-value) estimates and modules related to a variable were selected as those with a q-value < 0.05.

Every module that exhibited high correlation with a treatment or harboured many candidate lncRNAs was tested for enrichment of GO terms and KEGG pathways with gProfiler [61]. The list of all expressed genes was used as the statistical domain scope for the test and the significance threshold was set to 0.05 Benjamini-Hochberg FDR. Gene ontology term networks were created with the EnrichmentMap plugin workflow [62] for Cytoscape v3.7.1 [63] using enrichment results from gProfiler, and clusters of terms were formed by semantic similarity. Apart from enrichment analysis, the hub genes of each module were obtained by calculating the module membership (MM) and gene significance (GS) values according to WGCNA. We defined hub genes as those belonging to the ≥ 85th percentile for both MM and GS in each module. Those genes, including lncRNAs, are likely key drivers of expression and can give an idea about the functions or pathways of candidate lncRNAs in those modules.

### Correlations of nearby lncRNA-PCG pairs

Candidate lncRNA-PCG pairs for cis-regulation were obtained from expression correlations between closely located pairs. Candidate lncRNAs whose TSSs were located less than 100 kb apart from the TSS of another annotated gene were selected, and the Spearman correlation was calculated between the expression profiles of both genes. Pairs with an absolute correlation R higher than 0.8 and a FDR-corrected p-value lower than 0.05 were kept.

### Identification of potential miRNA sponges

MicroRNA expression data from the same experiment was downloaded from GEO (series GSE113897). RIsearch2.1 [64], a large-scale RNA–RNA interaction prediction tool suitable for full genome or transcriptome screening, was used to predict miRNA target sites in all the expressed transcripts. The minimum seed size was set to 6, the seed had to be within the first 8 bases of the miRNA and G-U wobbles were allowed, as proposed by the authors. Hybridization threshold was set to -15 kcal/mol. For a transcript to be classified as a potential miRNA sponge we set the minimum of 20 target sites of a single miRNA and the quantity of target sites in each transcript was averaged for visualisation at gene level. PCG, lncRNA and miRNA expression levels were normalised by TPM and Pearson correlations were performed between miRNAs and their putative sponge genes. Significant negative correlations were visualized with Cytoscape v3.7.1 [63].

### RT-qPCR experiments

The relative quantification of 10 lncRNAs and 10 PCGs was performed by RT-qPCR using 16 different animals, 4 from each treatment group. We chose a heterogeneous set of lncRNA-PCG pairs regarding DE status and relative position of the lncRNA. They were required to be correlated at gene expression level and less than 5 kb apart. Primers were designed using PrimerQuest and OligoAnalyzer tools of Integrated DNA Technologies (IDT) (Additional file 5). GAPDH, ATPase, ACTB and G6PD were used as putative reference genes. RT-qPCR experiment was carried out using BioMark HD Nanofluidic qPCR System technology (Fluidigm) combined with a GE 48.48 Dynamic Array integrated fluidic circuit (IFC) and the Master Mix SsoFast EvaGreen Supermix with Low ROX (Bio-Rad). RT-qPCR experiment was performed at the Gene Expression Unit of the Genomics Facility, in the General Research Services (SGIKER) of the UPV/EHU.

Analysis of amplification data was carried out using the Fludigm Real-Time PCR Analysis Software [4.1.3]. Amplification curves and melting curves were analysed to discard low quality amplifications and Ct values were corrected for efficiency differences with GenEx software of MultiD [5.4]. The stability of candidate reference genes was analysed with NormFinder and GeNorm, implemented in GenEx. G6PD and ACTB were the most stable reference genes. Relative quantification for the correlations between lncRNAs and PCGs were determined by the ΔCt method and log2 fold changes for the validation of differential expression of lncRNAs were calculated with the ΔΔCt method. Normal distribution was checked using the Shapiro-Wilk test, and because the null hypothesis was rejected, Spearman’s rank correlation coefficient was used to assess the presence of significant correlation and non-parametric tests for pairwise comparisons.

## Supplementary Information

**Additional file 1.** Annotation of the novel sheep lncRNAs described in this study.

**Additional file 2.** Conserved lncRNAs in each of the four species used for the analysis in terms of sequence and synteny.

**Additional file 3.** Results from the differential expression analysis in the three comparisons.

**Additional file 4.** Full results of the co-expression analysis: Co-expression modules, hub genes, eigengenes and gene set enrichment analysis of the co-expression modules.

**Additional file 5.** List of the selected protein coding genes and lncRNAs for the RT-qPCR analysis and their corresponding primer sequences.

## Data Availability

The datasets analysed during the current study are available in the GEO repository with accession number GSE113899, https://www.ncbi.nlm.nih.gov/geo/query/acc.cgi?acc=GSE113899. Custom python scripts used are available at https://github.com/bilbaom/vaccine-lncrnas-sheep.
